# How Social Power Affects the Processing of Angry Expressions: Evidence From Behavioral and Electrophysiological Data

**DOI:** 10.3389/fpsyg.2020.626522

**Published:** 2021-01-21

**Authors:** Entao Zhang, Xueling Ma, Ruiwen Tao, Tao Suo, Huang Gu, Yongxin Li

**Affiliations:** ^1^ Institute of Cognition, Brain and Health, Henan University, Kaifeng, China; ^2^ Institute of Psychology and Behavior, Henan University, Kaifeng, China

**Keywords:** angry faces, social power, event-related potentials, P1, P3

## Abstract

With the help of event-related potentials (ERPs), the present study used an oddball paradigm to investigate how both individual and target power modulate neural responses to angry expressions. Specifically, participants were assigned into a high-power or low-power condition. Then, they were asked to detect a deviant angry expression from a high-power or low-power target among a series of neutral expressions, while behavioral responses and electroencephalogram (EEG) were recorded. The behavioral results showed that high-power individuals responded faster to detect angry expressions than low-power individuals. The ERP analysis showed that high-power individuals showed larger P3 amplitudes in response to angry expressions than low-power individuals did. Target power increased the amplitudes of the P1, VPP, N3, and P3 in response to angry expressions did, but decreased the amplitudes of the N1 and N170 in response to angry expressions. The present study extended previous studies by showing that having more power could enhance individuals’ neural responses to angry expressions in the late-stage processes, and individuals could show stronger neural responses to angry expressions from high-power persons in both the early‐ and late-stage processes.

## Introduction

The accurate recognition of emotional states from others’ facial expressions is particularly important to coordinate social relationships (van Dijk et al., 2008; Yamagishi et al., 2012). Anger is frequently fueled by blameworthy behaviors of others in social interactions (Averill, 1982; Weber, 2004). Since angry expressions usually occur in social contexts, the processing of angry expressions should be influenced by the context in which they occur (Hess and Hareli, 2015; Hareli and David, 2017). The present research focused on whether and how social power affects the processing of angry expressions.

Social power is the fundamental dimension of social relationships and social life, it is generally defined as one’s capacity to influence others by controlling resources (Keltner et al., 2003). There is ample evidence that social power has a wide range of consequences for one’s thoughts and feelings (Guinote, 2017). Major power theories assume that social power leads to reduced processing of others’ emotions, as high-power individuals who control resources tend not to attend to others’ emotions (Keltner et al., 2003; Russell and Fiske, 2010; Magee and Smith, 2013). In line with this view, there is increasing evidence that high-power individuals are less accurate in recognizing others’ emotional expressions and prosodies (Anderson and Berdahl, 2002; Galinsky et al., 2006; Martin et al., 2012; Paulmann and Uskul, 2016; Uskul et al., 2016). For example, in a widely cited experiment by Galinsky et al. (2006, Study 3), participants were asked to recall and write about a situation in which they had power over another person (high-power condition), or recall and write about their previous day’s events (control condition). Following the power manipulation, they were asked to observe a series of faces and judge the emotional expressions with four response choices (i.e., happiness, fear, anger, or sadness). The results showed that high-power individuals were less accurate in judging emotional expressions than control participants did. Similarly, with the addition of a low-power condition, another study also found lower accuracy in emotional prosody recognition for high-power individuals when compared to low-power individuals (Blader et al., 2016).

In contrast, there is also conflicting evidence that high-power is associated with better performance at recognizing emotional expressions (Schmid Mast et al., 2009; Côté et al., 2011). For example, with the addition of a low-power condition, Schmid Mast et al. (2009, Study 3) replicated study of Galinsky et al. (2006). The results showed that high-power individuals performed more accurately than neutral and low-power participants did. The mixed nature of findings presents the possibility that there are moderators that affect the power-anger link. For example, Nissan et al. (2015) found that power impeded emotional recognition in female but not in male participants. In addition to the gender variable, there are other variables that affect both the direction and magnitude of the effect of individual power on emotional recognition.

The processing of emotional expressions is not only affected by individual power, but also biased by target power (emotion expresser; Ratcliff et al., 2012a,b; Carr et al., 2014). For example, in an emotion recognition task, participants were asked to identify the emotional expression (anger, fear, happiness, or neutral) from high‐ or low-status persons. The results showed that angry expressions were identified with greater accuracy when they appeared on high-status faces than low-status faces, but only for people who were high in social dominance orientation (Ratcliff et al., 2012a). In a subsequent study (Ratcliff et al., 2012b), participants were asked to see faces from high-or low-status persons, and to indicate when a face had shifted from an initial fear expression into an angry expression. The results showed that angry expressions appeared sooner on the faces of high-status compared to low-status targets. The authors concluded that target power could influence the perception of angry expressions. Recently, using the facial electromyography (fEMG) technique, it was shown that high-power individuals smiled more when they watched angry expressions from high-power targets than low-power targets. Instead, low-power individuals smiled equally to angry expressions from both high-power targets and low-power targets (Carr et al., 2014). In sum, these results suggest that the effects of individual power on emotional recognition appear to depend on target power. The responsive bias in high-power individuals might reflect that high-power individuals have a more flexible way in processing others’ emotions than low-power individuals. The results are consistent with the situated focus theory of power (Guinote, 2007a,b), which assumes that having power enhances one’s ability to focus their attention on relevant information and use relevant contextual information in constructing social meanings.

Different from theories, which predict that high-power individuals are less accurate in processing others’ emotions than low-power individuals (Keltner et al., 2003; Magee and Smith, 2013), the situated focus theory of power suggests that high-power individuals react in a flexible way to others’ emotional expressions. Specifically, high-power individuals do not always exhibit better performance in recognizing others’ emotional expressions. High-power individuals’ performance is often better in complex and stressful tasks, as their motivation to complete tasks will be elicited only when they are under threat. In contrast, low-power individuals are argued to be less able to use relevant contextual information in constructing social reactions, as they are always under threat (Guinote, 2017). According to the view of the situated focus theory of power, the links between individual power and performance in facial emotion recognition might depend on task difficulty and social importance of others’ emotional expressions, as these factors can modulate high-power individuals’ motivation to perform tasks.

Taken together, although accumulative evidence has demonstrated that the recognition of emotional expressions is modulated by both individual and target power, no study so far has simultaneously manipulated both individual and target power to examine the effects of power on the recognition of emotional expressions. Therefore, we will use event-related potentials (ERPs) to investigate how did both individual and target power modulate neural responses to angry expressions. The reason for selecting angry expressions as target stimuli is that angry expressions are important signals of both danger and threat, and have a high incentive value for high-power individuals. Besides, as mentioned above, anger expressed by high-power people are especially salient for individuals with high social dominance orientation (Ratcliff et al., 2012a). Thus, in our study, we presented participants with angry expressions appearing on high-power or low-power targets. We hypothesized that high-power individuals tend to perform better performance in detecting angry expressions from high-power targets than low-power targets, as attention to angry expressions from high-power targets are be important for high-power individuals. In contrast, we hypothesized that low-power individuals tend to perform equal performance in detecting angry expressions whether they appear on high-power targets or low-power targets, as the threat signaled by angry expressions is always relevant for them.

Besides, the present study used a modified oddball paradigm that required subjects to make a standard/non-target deviant/target-deviant distinction by pressing the key. Rather than requiring participants to select an emotional word to match observed emotional expressions in prior studies (Galinsky et al., 2006), the modified oddball task has two advantages. Firstly, it should be noted that participants are always under time pressure to complete the modified oddball task, consequently to allow for investigating high-power individuals’ performance in stressful tasks (Wang et al., 2014; Guinote, 2017). Secondly, both response accuracy and time can be used as indicators to examine the effect of power on emotional recognition.

Another limitation in previous studies is that the behavioral method cannot assess the different stages of neural responses to angry expressions. It remains unclear the exact time course of the effects of both individual and target power on the processing of angry expressions. ERPs can help elucidate this issue due to its excellent temporal resolution. Previous ERP studies have shown that various ERP components can be related to the processing of emotional expressions (see reviews in Eimer and Holmes, 2007). For example, P1, N1, N170, and VPP are considered to reflect the early phase of perception and attention processing of emotional stimuli, whereas N3 and P3 are considered to reflect the later phase of emotional discrimination and evaluation (e.g., Eimer and Holmes, 2007; Luo et al., 2010; Rellecke et al., 2012).

Specifically, the occipital P1 component (with a 100–130 ms peak latency) and superior parietal N1 (with a 100–150 ms peak latency) have been enhanced for negative relative to neutral expressions (Santesso et al., 2008; Houston et al., 2018). Enhancement of the P1 and N1 has been linked to the amplification of initial visuospatial attention to threat-related stimuli (Vuilleumier, 2005; Rellecke et al., 2012). Following P1, the occipital temporal N170 (Peaks around 170 ms) has been associated with rapid stages of structural encoding of faces (Bentin et al., 1996). It has been reported that the N170 is enhanced for emotional relative to neutral expressions (Blau et al., 2007; Eimer and Holmes, 2007; Hinojosa et al., 2015). Also, the frontocentral VPP (with a latency similar to that of the N170) is related to the configural processing of faces (Eimer, 2000). Some studies have also found augmented VPP for emotional stimuli (Williams et al., 2006; Luo et al., 2010).

In the mid-latency range, the central N300 (Peaks around 200–350 ms) has been found to be sensitive to neutral, positive, and negative expressions (Ashley et al., 2004; Williams et al., 2006). Thus N300 might involve affective discrimination (Calvo and Beltrán, 2013). In the long-latency range, the frontocentral P3 is also reactive to facial expressions (e.g., Luo et al., 2010; Rellecke et al., 2012). P3 might involve higher-level phases of stimulus evaluation and selection and is a broad index of the strength of approach-avoidance motivation (Lang and Bradley, 2010; Luo et al., 2010; Calvo and Beltrán, 2013). Some studies indicated that individuals’ need for power enhances the P3 amplitudes in the processing of angry expression (Wang et al., 2014; Paulmann and Uskul, 2016). For example, using an ERP oddball paradigm, Wang et al. (2014) asked participants with high‐ and low‐ need for power to detect, among a series of standard stimuli (neutral faces), an infrequent angry face varying on anger intensity (50, 100, or 150%). The results showed that high-intensity (150%) anger expressions elicited larger P3 amplitudes relative to prototypical (100%) anger expressions for individuals with high-need for power, but not for individuals with low-need for power. These findings suggest that the need for power modify the later emotional evaluation stage of processing angry expressions.

With the help of ERPs, the present study investigated how both individual and target power modulates neural responses to angry expressions. Participants were assigned into a high-power/low-power group, and were primed by power episodes. Then, they were asked to detect, among a series of neural expressions, a deviant angry expression from a high-power or low-power target, while behavioral responses and electroencephalogram (EEG) were recorded. Using this method, we sought to clarify at which stages of processing angry expressions both individual and target power may alter face processing. More specifically, we investigated whether both individual and target power already modifies the early attentional processing (i.e., P1 and N1) and structural encoding (i.e., N170 and VPP) of processing angry expressions and whether possible modulations occur at the later emotional evaluation stage (i.e., N3 and P3).

With respect to neural correlates, three hypotheses were formulated as follows: (1) according to previous findings that neural responses to angry expressions are modulated by the need for at the relative later stage (i.e., P3 or LPC; Wang et al., 2014), if the effect of individual power on processing angry expressions could occur at the later emotional evaluation stage, then the N3 and P3 amplitudes elicited by angry expressions would be larger for high-power individuals than for low-power individuals; (2) according to the view that target power could influence the perception of angry expressions (Ratcliff et al., 2012b), if the effects of target power on processing angry expressions could occur at the early attentional and perceptual processing stages, then the early components (i.e., P1, N1, N170, and VPP) elicited by angry expressions would be enhanced in the high-power target condition than in the low-power target condition. In contrast, if target power could modify both the early attentional and perceptual processing and later emotional meaning evaluation of angry expressions, the early components (i.e., P1, N1, N170, and VPP) and later components (i.e., N3 and P3) would be modulated simultaneously; (3) based on the view of the situated focus theory of power (Guinote, 2007a,b) that high-power individuals tend to pay attention to angry expressions from high-power targets than low-power targets, while the attention bias is absent in low-power individuals, the interaction of both power factors would be observed in neural correlates. Because, we did not know how exactly these components would be influenced by our experimental manipulations, no clear *a priori* hypothesis was formulated.

## Materials and Methods

### Participants

Forty-four undergraduate students (22 female, 22 male; mean age 21.9 years) recruited from Henan University were randomly assigned to a high-power or low-power condition, 22 each in the high-power (12 male) and low-power groups (10 male). Sample size was determined on the basis of previous ERP studies exploring the difference between high‐ and low-power individuals (e.g., Paulmann and Uskul, 2016). Besides, the data from four participants were discarded due to intensive head movements during EEG recording. Finally, 40 participants’ data (20 each in the high-power and low-power groups) were included in both the behavioral and ERP analyses. All participants had a normal or corrected-to-normal vision and they were all right-handed. Also, they reported no history of affective disorder and were free of any psychiatric medication. This study was conducted under the guidelines of the Declaration of Helsinki and approved by the local Ethics Committee of Henan University. All participants signed informed consent before the experiment.

### Materials

FaceGen Modeler software (version 3.4, Singular Inversions, 2009) is a software package that has been widely used to create emotional faces in multiple investigations (Said et al., 2010; Recio et al., 2011; Wilkowski and Robinson, 2012). This software uses a large database of faces to generate faces that are realistic in appearance. Using this program four southeast Asian male faces were generated as original material for the experiment. Afterward, the age and the emotion of each of the four faces were varied to create three faces: a 43-years-old neutral face, a 43-years-old angry face, and a 65-years-old neutral face. The angry expression was 100 percent intense. Consequently, there were 4 × 3 = 12 faces. All the stimuli were presented in the center of the screen with an exposure duration of 1,000 ms, and a visual angle of 3.68° × 3.42° from a viewing distance of 70 cm. The stimulus materials were divided into standard stimuli and deviant stimuli. The standard stimuli were the 43-years-old neutral expression faces, and the deviant stimuli included both the 43-years-old angry faces and the 65-years-old neutral faces.

### Procedure

Firstly, we manipulated individual power by assigning them to complete a high-power or low-power writing prime (Galinsky et al., 2003). Specifically, the participants were asked to recall and describe a particular incident in which they had power over another individual (high power prime) or someone else had power over them (low power prime) within 15 min. According to the research of Kraus et al. (2011), the manipulation check of individuals power was conducted by asking participants to respond to a two-item question: “now I feel I have a great sense of power” and “now I feel my wishes are not important” (reverse scoring). Responses were made using seven-point Likert scales (1 = “strongly disagree,” 7 = “strongly agree”; *r* = 0.89). The manipulation check confirmed that participants in the high power condition (*M* = 5.00, *SD* = 1.15) rated themselves as more powerful than those in the low power condition (*M* = 3.90, *SD* = 0.84), *t*(38) = −3.47, *p* = 0.001.

Secondly, target power was manipulated according to previous studies (Ratcliff et al., 2012b; Carr et al., 2014). Target power was manipulated by randomly pairing one target face with a high-power occupational profile (e.g., President of a university or Dean of a Faculty, these occupations represent high status or power) or a low-power occupational profile (e.g., Cleaners or Mechanic, these occupations represent low status or power). To balance the link between face and power, the same faces were associated with opposite social status information among participants.

**Figure 1:**
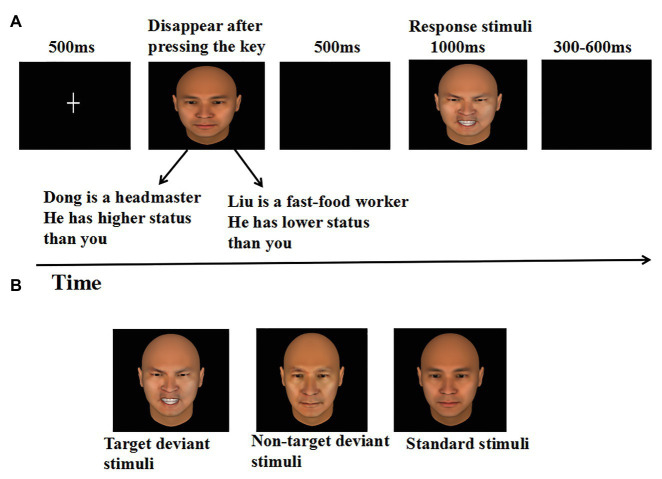
**(A)** Sequence of events in a representative trial of the experiment, and **(B)** the example stimuli.

Thirdly, participants were instructed to complete an emotional detection task in the oddball paradigm. The oddball task consisted of four blocks, each containing 100 trials. Each block contained three versions of the same person’s face, including a 43-years-old neutral expression face for standard stimulus (70%), a 43-years-old angry face (target), and a 65-years-old neutral face (non-target) for deviant stimulus (15% respectively). As illustrated in Figure 1. Each trial began with a fixation point (“+”) for 500 ms at the center of the black computer screen. Immediately after fixation point offset, participants were informed that they would see a face from different occupational profiles, which was displayed at the center of the screen, and remained in view until a response was given, or until 4 s had passed. Each occupational profile was paired with one person’s face and emphasized the person’s high or low power relative to the participant. The numbers of high‐ and low-power target trials were equal. Then, after a blank screen for 500 ms, a face was pseudorandomly presented at the center of the screen for 1 s. Participants were asked to ignore the standard stimuli (43-years-old neutral expression faces) and the non-target deviant stimuli (65-years-old neutral faces), and were asked to press the “F” key on the keyboard with the left index finger as accurately and quickly as possible if the target deviant stimuli (43-years-old angry faces) appeared. Each response was followed by a random delay of 300–600 ms blank screen. It has to be pointed out that in each block 10 standard stimuli were presented in the head of the sequence to establish sensory memory pattern, and there were no less than two standards between consecutive deviants (Xu et al., 2013).

Lastly, the method by Blue et al. (2018) was used to check target manipulation. At the ending of each block, the participants were asked to indicate to what extent they felt the target’s power on a seven-point Likert scale (1 = “very inferior”; 7 = “very superior”). A mixed-model ANOVA with target power as the dependent variable, type of target status as a within-subjects factor, and individual power prime as a between-subject factor was conducted. There was no significant main effect of individual power, *F* (1, 38) = 1.81, *p* = 0.19. The main effect of target power was significant, *F* (1, 38) = 98.98, *p* < 0.001, *η* ^2^ = 0.723, suggesting that the high-status targets were rated more powerful (*M* = 5.11, *SD* = 0.07) than the low status target (*M* = 3.40, *SD* = 0.14). The interaction between individual power and target power was not significant, *F* (1, 38) = 1.187, *p* = 0.283.

### Electrophysiological Recording and Analysis

Brain electrical activity was recorded from 32 scalp sites using electrodes mounted on an Ag/AgCl cap (Brain Product), with the reference on the left and right mastoids, EEG signal was recorded from electrodes arranged according to the standard 10–20 system. The vertical electrooculogram (EOG) was recorded with electrodes placed above and below the right eye. All inter-electrode impedance was maintained below 5 kΩ. The EEG and EOG were amplified using a 0.01–30 Hz bandpass and continuously sampled at 500 Hz/channel.

Brain Vision Analyzer software (Brain Products) was used for off-line analysis. The Independent Component Analysis (ICA) was employed to remove ocular artifacts. All epochs in which EEG voltages exceeded a threshold of ±80 μV were excluded from further processing. There were more than 50 effective trials (the *SD* range is between 2 and 4) remained for each condition in each participant. The EEG was time-locked to the onset of angry faces and was segmented into the epoch from 200 ms pre-stimulus to 1,000 ms post-stimulus.

Based on the topographical distribution of grand-averaged ERP activity and previous studies (Luo et al., 2010; Rellecke et al., 2012), we selected the time windows and electrode sites of six ERP components: P1, N1, N170, VPP, N300, and P300. The following five electrode sites (O1, O2, Pz, P3, and P4) were selected for statistical analysis of the P1 component (100–130 ms); Fz, F3, F4, FC1, and FC2 were selected for statistical analysis of the N1 component (90–130 ms); VPP (150–180 ms) were analyzed at the Fz, F3, F4, C3, C4, Cz, FC1, and FC2 electrode sites; N170 (150–180 ms) was analyzed at the P7, P8 electrode sites; and N3 (250–350 ms) was analyzed at the T7, T8 electrode sites. Fz, F3, F4, C3, C4, Cz, Pz, P3, P4, P7, P8, FC1, FC2, FC5, FC6, CP1, CP2, CP5, and CP6 were selected for statistical analysis of the P3 component (380–520 ms). The averaged amplitudes were analyzed for each component. A mixed model ANOVA on the amplitude of each component was conducted, value of *p* were corrected by Greenhouse–Geisser correction.

## Results

### Behavioral Data

The behavioral data analysis were only on the target trials. After inspecting the data in accuracy rates, we found approximately 100% accuracy on all trial types (the range of accuracy rates was between 97 and 99%). Because there was not enough variability in response errors to conduct meaningful analyses, and accuracy rates were discarded from further analysis.

**Figure 2:**
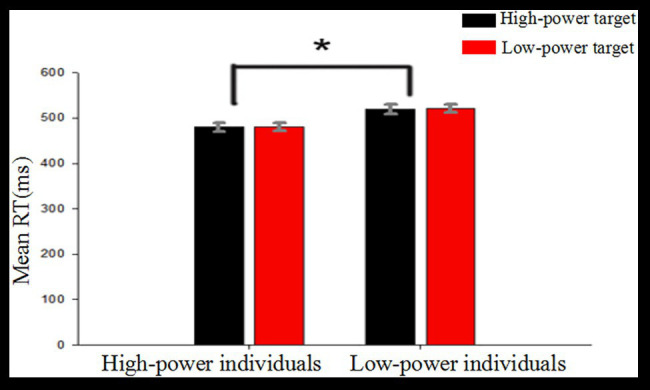
Descriptive statistics for response times (ms) for each condition. ^*^ *p* < 0.05.

A mixed-model ANOVA with average reaction time as the dependent variable, type of target power as a within-subject factor, and individual power as a between-subjects factor revealed a significant main effect of individual power, *F* (1, 38) = 8.24, *p* = 0.007, *η* ^2^ = 0.178. *Post hoc* analysis revealed that average RTs were shorter for high-power individuals (*M* = 481, *SD* = 9) as compared to low-power individuals (*M* = 520, *SD* = 9). The main effect of target power was not significant, *F* (1, 38) = 0.19, *p* = 0.663. The interaction between individual power and target power was not significant, *F* (1, 38) = 0.015, *p* = 0.905 (see Figure 2).

### ERP Data

#### P1

**Figure 3:**
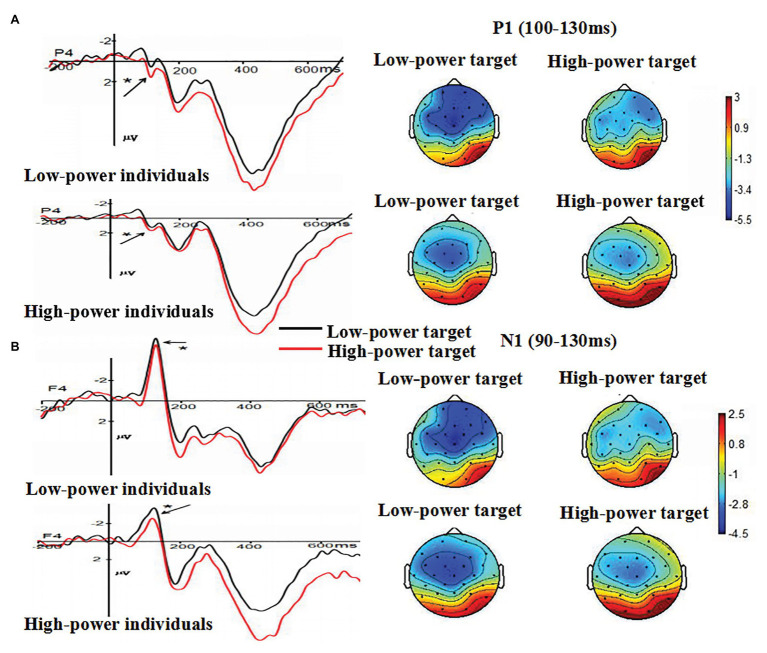
**(A)** The Grand-average P1 as a function of individual power (high vs. low) and target power (high vs. low) at P4 electrode site, and **(B)** the Grand-average N1 as a function of individual power (high vs. low) and target power (high vs. low) at F4 electrode site. Topographical maps for all conditions were presented. ^*^ *p* < 0.05.

The main effect of individual power was not significant, *F* (1, 38) = 0.691, *p* = 0.411. The main effect of target power was significant, *F* (1, 38) = 5.00, *p* = 0.031 (*p* < 0.05), *η* ^2^ = 0.116, suggesting that angry expressions from high-power targets (*M* = 0.915 μV, *SE* = 0.46) elicited larger amplitudes than angry expressions from low-power targets (*M* = 0.081 μV, *SE* = 0.47) did. The main effect of electrode was significant, *F* (4, 152) = 23.277, *p* < 0.001, *η* ^2^ = 0.380, suggesting the largest P1 amplitudes on O_2_ (*M* = 2.008 μV, *SE* = 0.55). The interaction between individual power and target power was not significant *F* (1, 38) = 0.010, *p* = 0.92 (*p* > 0.05). The interaction between individual power and electrode was not significant, *F* (4, 152) = 0.317, *p* = 0.73 (*p* > 0.05). The interaction between target power and electrode was not significant, *F* (4, 152) = 1.505, *p* = 0.22 (*p* > 0.05). These findings indicated that anger expressed by high-power persons could increase individuals’ early attention processing, as indexed by the P1 component (see Figure 3A).

#### N1

The main effect of individual power was not significant, *F* (1, 38) = 0.02, *p* = 0.899 (*p* > 0.05). The main effect of target power was significant, *F* (1, 38) = 6.563, *p* = 0.015 (*p* > 0.05), *η* ^2^ = 0.147. Compared with N1 amplitudes induced by angry expressions from high-power targets (*M* = −2.272 μV, *SE* = 0.48), larger N1 amplitudes were induced by angry expressions from low-power targets (*M* = −3.43 μV, *SE* = 0.52). The main effect of electrode was not significant, *F* (4, 152) = 1.109, *p* = 0.324 (*p* > 0.05). The interaction between individual power and target power was not significant, *F* (1, 38) = 0.484, *p* = 0.491 (*p* > 0.05). The interaction between individual power and electrode was not significant, *F* (4, 152) = 0.805, *p* = 0.423 (*p* > 0.05). The interaction between target power and electrode was not significant, *F* (4, 152) = 1.477, *p* = 0.230 (*p* > 0.05). These findings indicated that anger expressed by high-power persons could increase individuals’ attention processing, indexed by the N1 component (see Figure 3B).

#### N170

**Figure 4:**
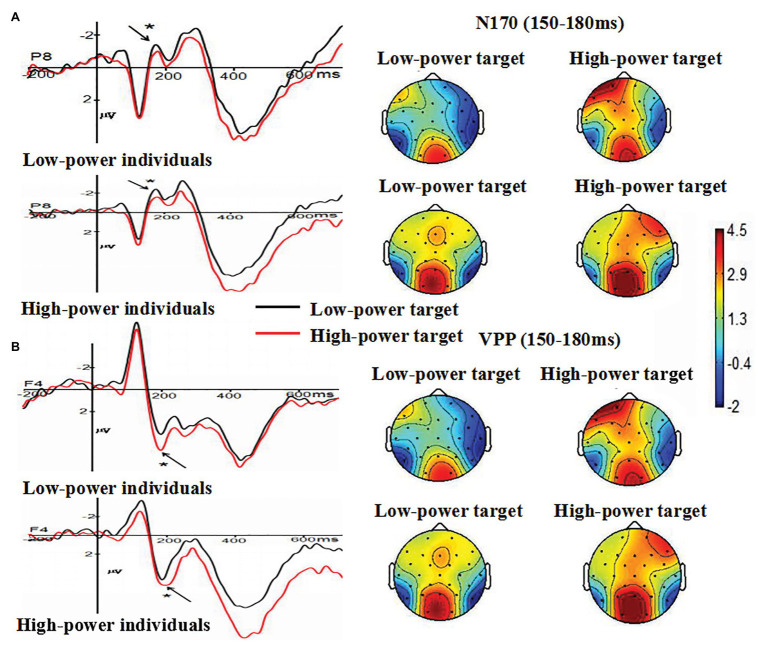
**(A)** The Grand-average N170 as a function of individual power (high vs. low) and target power (high vs. low) at P8 electrode site, and **(B)** the Grand-average VPP as a function of individual power (high vs. low) and target power (high vs. low) at F4 electrode site. Topographical maps for all conditions were presented. ^*^ *p* < 0.05.

The main effect of individual power was not significant, *F* (1, 38) = 0.002, *p* = 0.966 (*p* > 0.05). The main effect of target power was significant, *F* (1, 38) = 4.713, *p* = 0.036 (*p* < 0.05), *η* ^2^ = 0.110. Compared with N170 amplitudes induced by angry expressions from high-power targets (*M* = −0.654 μV, *SE* = 0.441), larger N170 amplitudes were induced by angry expressions from low-power targets (*M* = −1.436 μV, *SE* = 0.452). The main effect of electrode was not significant, *F* (1, 38) = 0.025, *p* = 0.876 (*p* > 0.05). The interaction between individual power and target power was not significant, *F* (1, 38) = 0.023, *p* = 0.880 (*p* > 0.05). The interaction between individual power and electrode was not significant, *F* (1, 38) = 0.823, *p* = 0.370 (*p* > 0.05), The interaction between target power and electrode was not significant, *F* (1, 38) = 0.764, *p* = 0.388 (*p* > 0.05). These findings indicated that anger expressed by high-power persons could decrease individuals’ processing of structural encoding of angry expressions, as indexed by N170 (see Figure 4A).

#### VPP

The main effect of individual power was not significant, *F* (1, 38) = 0.154, *p* = 0.697 (*p* > 0.05). The main effect of target power was significant, *F* (1, 38) = 4.135, *p* = 0.049 (*p* < 0.05), *η* ^2^ = 0.098. Compared with VPP amplitudes induced by angry expressions from low-power targets (*M* = 1.374 μV, *SE* = 0.873), larger VPP amplitudes were induced by angry expressions from high-power targets (*M* = 2.344 μV, *SE* = 1.065). The main effect of electrode was not significant, *F* (7, 266) = 1.282, *p* = 0.284 (*p* > 0.05). The interaction between individual power and target power was not significant, *F* (1, 38) = 1.667, *p* = 0.204 (*p* > 0.05). The interaction between individual power and electrode was not significant, *F* (7, 266) = 0.573, *p* = 0.615 (*p* > 0.05). The interaction between target power and electrode was not significant, *F* (7, 266) = 0.584, *p* = 0.664 (*p* > 0.05). These findings indicated that anger expressed by high-power persons could increase individuals’ processing of structural encoding of angry expressions, as indexed by VPP (see Figure 4B).

#### N3

**Figure 5:**
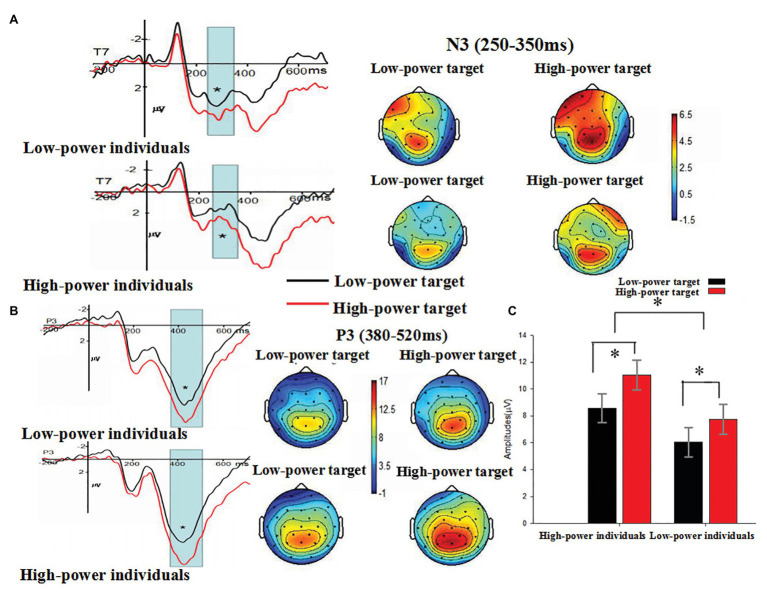
**(A)** The Grand-average N3 as a function of individual power (high vs. low) and target power (high vs. low) at T7 electrode site, and **(B)** the Grand-average VPP as a function of individual power (high vs. low) and target power (high vs. low) at P3 electrode site. The time windows used for testing N3 (250–350 ms) and P3 (380–520 ms) were marked by gray bars. Topographical maps for all conditions were presented. **(C)** The means and SE (μV) of P3 component at P3 electrode site for each condition. ^*^ *p* < 0.05.

The main effect of individual power was not significant, *F* (1, 38) = 0.087, *p* = 0.770 (*p* > 0.05). The main effect of target power was significant, *F* (1, 38) = 4.674, *p* = 0.037 (*p* < 0.05). Compared with N3 amplitudes induced by angry expressions from low-power targets (*M* = 1.317 μV, *SE* = 0.544), larger N3 amplitudes were induced by angry expressions from high-power targets (*M* = 2.433 μV, *SE* = 0.517). The main effect of electrode was significant, *F* (1, 38) = 5.638, *p* = 0.023 (*p* < 0.05), *η* ^2^ = 0.129, suggesting the largest N3 amplitudes on T7 (*M* = 2.988 μV, *SE* = 0.668). The interaction between individual power and target power was not significant, *F* (1, 38) = 0.215, *p* = 0.646 (*p* > 0.05). The interaction between individual power and electrode reached marginal significance, *F* (1, 38) = 3.545, *p* = 0.067, *η* ^2^ = 0.085. Further analysis showed that there was no significant difference between high and low power participants at T7, *t*(38) = 1.112, *p* = 0.273 and T8, *t*(38) = −1.573, *p* = 0.124. The interaction between target power and electrode was not significant, *F* (1, 38) = 0.033, *p* = 0.856 (*p* > 0.05). These findings indicated that anger expressed by high-power persons could increase individuals’ processing of emotional evaluation of angry expressions, as indexed by N3 (see Figure 5A).

#### P3

The main effect of individual power was significant, *F* (1, 38) = 4.251, *p* = 0.046 (*p* < 0.05), *η* ^2^ = 0.101, suggesting that high-power individuals showed larger P3 amplitudes (*M* = 9.798 μV, *SE* = 0.992) than low-power individuals (*M* = 6.906 μV, *SE* = 0.992) did. The main effect of target power was also significant, *F* (1, 38) = 9.798, *p* = 0.003 (*p* < 0.01), *η* ^2^ = 0.205. Compared with P3 amplitudes induced by angry expressions from low-power targets (*M* = 7.307 μV, *SE* = 0.766), larger P3 amplitudes were induced by angry expressions from high-power targets (*M* = 9.396 μV, *SE* = 0.787). The main effect of electrode was significant, *F* (18, 684) = 24.940, *p* < 0.001, *η* ^2^ = 0.396, suggesting the largest P3 amplitudes on P_Z_ (*M* = 14.019 μV, *SE* = 1.015). The interaction between individual power and target power was not significant, *F* (1, 38) = 0.308, *p* = 0.582 (*p* > 0.05). The interaction between individual power and electrode was not significant, *F* (18, 684) = 0.211, *p* = 0.949 (*p* > 0.05). The interaction between target power and electrode was not significant, *F* (18, 684) = 1.442, *p* = 0.215 (*p* > 0.05), *η* ^2^ = 0.037. The results showed P3 amplitudes were enhanced by both individual power and target power (see Figures 5B,C).

## Discussion

Using an ERP oddball paradigm, combined with a high temporal resolution of ERP technology, the present study examined cerebral sensitivity to angry faces from high-power and low-power targets between high-power and low-power individuals. To our knowledge, ERP patterns of simultaneously manipulating both individual and target power have not been investigated before. Behaviorally, our results showed that high-power individuals responded faster to detect angry expressions than low power individuals did. At the electrophysiological level, for individual power, high-power individuals showed larger P3 amplitudes to angry expressions than low-power individuals did. For target power, compared with angry expressions from low-power target, angry expressions from high-power targets elicited larger P1, VPP, N3, and P3 amplitudes, but induced smaller N1 and N170 amplitudes. Moreover, the interaction effect between individual power and target was not found in both the behavioral and EEG results. Next, we will discuss the implications of these findings from both behavioral and EPR measures.

Our findings enhanced our understanding of the mixed evidence on how individual power is related to facial emotion recognition. There has been a debate on whether individual power facilitates or hinders emotional face recognition. A large amount of evidence has shown some positive, negative, and null associations between individual power and emotional recognition accuracy (e.g., Galinsky et al., 2006; Schmid Mast et al., 2009; Hall et al., 2014; Nissan et al., 2015). In an ERP oddball task, we found that having more power enhanced individuals’ sensitivity to angry expression in both the behavioral and ERP data. In the behavioral results, high-power individuals responded faster to detect angry expressions than low power individuals did. In the ERP results, high-power individuals showed larger P3 amplitudes to angry expressions than low-power individuals did. As has been established, P3 signals the later phase of meaning evaluation of emotional stimuli, in which the significance of emotional information is analyzed and evaluated more fully (Luo et al., 2010; Calvo and Beltrán, 2013). In addition, P3 is also assumed to be a broad index of the strength of approach-avoidance motivation, as emotional stimuli with increased significance elicit more attentional resources and stronger approach or avoidance motivation (Lang and Bradley, 2010). Together with the behavioral finding that faster responses to angry expressions in high-power individuals, we speculated that having more power could enhance individuals’ emotional evaluation and approach motivation to angry expressions. However, how did we explain the differences between our findings and previous findings (Galinsky et al., 2006; Nissan et al., 2015; Uskul et al., 2016)? One possible explanation is that our modified oddball task might increase individuals’ motivation to complete it, as some studies indicate that high power is usually found to enhance behavioral performance in complex tasks (e.g., Guinote, 2007a,b, 2017; Smith et al., 2008; Schmid et al., 2015). Besides, future studies should focus on the effects of individual power on emotional face recognition in different tasks.

Secondly, the effects of target power on processing angry expressions were not confirmed in the behavioral data. However, for the first time, the present study also provided electrophysiological evidence that the amplitudes of the P1, N1, N170, VPP, N3, and P3 components were modulated by target power. As mentioned in the introduction section, the P1, N1, N170, and VPP components are considered to reflect the early phase of sensory and perceptual processing of emotional stimuli, whereas N3 and P3 are considered to reflect the later phase of emotional discrimination and evaluation (Eimer and Holmes, 2007; Luo et al., 2010; Rellecke et al., 2012). Thus, we inferred that target power affected angry face processing at both the sensory processing and higher-level cognitive processing. Specifically, relative to P1 amplitudes elicited by angry expressions from low-power targets, larger P1 amplitudes were elicited by angry expressions from high-power targets, suggesting that target power could enhance individuals’ initial attention to angry expressions. Similarly, the larger VPP amplitudes in processing angry expressions from high-power targets suggested that target power could enhance individuals’ configural processing of angry expressions. In the later P3 stage, the larger P3 amplitudes in processing angry expressions from high-power targets suggested that target power could enhance individuals’ emotional evaluation processing of angry expressions. Besides, target power decreased the amplitudes of the N1 and N170. The reverse pattern in the N1 component between high‐ and low-power targets could be due to the fact that more attentional resources were allocated in P1 stage of processing angry expressions, which might hinder attention allocation in the N1 stage, as these two components were initiated at similar time points (Fu et al., 2005). Similarly, larger VPP might interfere with the processing of N170, thereby leading to smaller N170, as these two components are derived from the same neural dipole (Joyce and Rossion, 2005; Hofman et al., 2013).

Finally, several limitations of this research should be noted. Firstly, no interactions of individual power and target power on processing angry expression were found in both the behavioral and ERP data, this might be due to the disadvantageous method of power manipulations, a interpersonal interactive context would be used to manipulate power in future studies. Secondly, without other emotional conditions, there is reason to doubt that the modulated effect of power (individual/target) on anger expression processing might also be found in processing other expressions, which would make the results less convinced. The modulated effect of power (individual/target) on other emotional expressions will be tested in future.

## Conclusion

Despite these limitations, our results showed that having power facilitates angry emotion recognition. Moreover, the present findings provided further electrophysiological evidence that the P3 amplitudes elicited by viewing angry faces were modulated by individual power, whereas the amplitudes of the P1, N1, N170, VPP, N3, and P3 components elicited by viewing angry faces were modulated by target power. In conclusion, having more power could enhance individuals’ neural responses to angry expressions in the late-stage processes, and individuals could show stronger neural responses to angry expressions from high-power persons in both the early‐ and late-stage processes.

## Data Availability Statement

The raw data supporting the conclusions of this article will be made available by the authors, without undue reservation.

## Ethics Statement

The studies involving human participants were reviewed and approved by the local Ethics Committee of Henan University. The patients/participants provided their written informed consent to participate in this study.

## Author Contributions

EZ: writing-original draft. XM: formal analysis. RT and TS: data curation. HG and YL: writing – review and editing. All authors contributed to the article and approved the submitted version.

### Conflict of Interest

The authors declare that the research was conducted in the absence of any commercial or financial relationships that could be construed as a potential conflict of interest.

## References

[ref1] Anderson C. Berdahl J. L. (2002). The experience of power: examining the effects of power on approach and inhibition tendencies. J. Pers. Soc. Psychol. 83, 1362–1377. 10.1037/0022-3514.83.6.1362, PMID: 12500818

[ref2] Ashley V. Vuilleumier P. Swick D. (2004). Time course and specicity of event-related potentials to emotional expressions. Neuroreport 15, 211–216. 10.1097/00001756-200401190-00041 15106860

[ref3] Averill J. R. (1982). Anger and aggression: An essay on emotion. New York: Springer.

[ref4] Bentin S. Allison T. Puce A. Perez E. McCarthy G. (1996). Electrophysiological studies of face perception in humans. J. Cogn. Neurosci. 8, 551–565. 10.1162/jocn.1996.8.6.551, PMID: 20740065 PMC2927138

[ref5] Blader S. L. Shirako A. Chen Y. R. (2016). Looking out from the top: differential effects of status and power on perspective taking. Personal. Soc. Psychol. Bull. 42, 723–737. 10.1177/0146167216636628, PMID: 27036500

[ref6] Blau V. C. Maurer U. Tottenham N. McCandliss B. D. (2007). The face-specific N170 component is modulated by emotional facial expression. Behav. Brain Funct. 3:7. 10.1186/1744-9081-3-7, PMID: 17244356 PMC1794418

[ref7] Blue P. R. Hu J. Zhou X. L. (2018). Higher status honesty is worth more: the effect of social status on honesty evaluation. Front. Psychol. 9:350. 10.3389/fpsyg.2018.00350, PMID: 29615948 PMC5869916

[ref8] Calvo M. G. Beltrán D. (2013). Recognition advantage of happy faces: tracing the neurocognitive processes. Neuropsychologia 51, 2051–2061. 10.1016/j.neuropsychologia.2013.07.010, PMID: 23880097

[ref9] Carr E. W. Winkielman P. Oveis C. (2014). Transforming the mirror: power fundamentally changes facial responding to emotional expressions. J. Exp. Psychol. Gen. 143, 997–1003. 10.1037/a0034972, PMID: 24219023

[ref10] Côté S. Kraus M. W. Cheng B. H. Oveis C. van der Löwe I. Lian H.. (2011). Social power facilitates the effect of prosocial orientation on empathic accuracy. J. Pers. Soc. Psychol. 101, 217–232. 10.1037/a0023171, PMID: 21463075

[ref11] Eimer M. (2000). Effects of face inversion on the structural encoding and recognition of faces. Evidence from event-related brain potentials. Cogn. Brain Res. 10, 145–158. 10.1016/s0926-6410(00)00038-0, PMID: 10978702

[ref12] Eimer M. Holmes A. (2007). Event-related brain potential correlates of emotional face processing. Neuropsychologia 45, 15–31. 10.1016/j.neuropsychologia.2006.04.022, PMID: 16797614 PMC2383989

[ref13] Fu S. Caggiano D. M. Greenwood P. M. Parasuraman R. (2005). Event-related potentials reveal dissociable mechanisms for orienting and focusing visuospatial attention. Cogn. Brain Res. 23, 341–353. 10.1016/j.cogbrainres.2004.11.01, PMID: 15820641 PMC2366196

[ref14] Galinsky A. D. Gruenfeld D. H. Magee J. C. (2003). From power to action. J. Pers. Soc. Psychol. 85, 453–466. 10.1037/0022-3514.85.3.453, PMID: 14498782

[ref15] Galinsky A. D. Magee J. C. Inesi M. E. Gruenfeld D. H. (2006). Power and perspectives not taken. Psychol. Sci. 17, 1068–1074. 10.1111/j.1467-9280.2006.01824.x 17201789

[ref16] Guinote A. (2007a). Power affects basic cognition: increased attentional inhibition and flexibility. J. Exp. Soc. Psychol. 43, 685–697. 10.1016/j.jesp.2006.06.008

[ref17] Guinote A. (2007b). Behaviour variability and the situated focus theory of power. Eur. Rev. Soc. Psychol. 18, 256–295. 10.1080/10463280701692813

[ref18] Guinote A. (2017). How power affects people: activating, wanting, and goal seeking. Annu. Rev. Psychol. 68, 353–381. 10.1146/annurev-psych-010416-044153, PMID: 27687123

[ref19] Hall J. A. Schmid Mast M. Latu I. -M. (2014). The vertical dimension of social relations and accurate interpersonal perception: a meta-analysis. J. Nonverbal Behav. 39, 131–163. 10.1007/s10919-014-0205-1

[ref20] Hareli S. David S. (2017). The effect of reactive emotions expressed in response to another’s anger on inferences of social power. Emotion 17, 717–727. 10.1037/emo0000262, PMID: 28080088

[ref21] Hess U. Hareli S. (2015). “The role of social context for the interpretation of emotional facial expressions” in Understanding facial expressions in communication. eds. Mandal M. K. Awasthi A. (New York: Springer), 119–141.

[ref22] Hinojosa J. A. Mercado F. Carretié L. (2015). N170 sensitivity to facial expression: a meta-analysis. Neurosci. Biobehav. Rev. 55, 498–509. 10.1016/j.neubiorev.2015.06.002, PMID: 26067902

[ref23] Hofman D. Terburg D. van Wielink L. Schutter D. J. L. G. (2013). Coalescence of dominance motivation and responses to facial anger in resting-state and event-related electrophysiology. NeuroImage 79, 138–144. 10.1016/j.neuroimage.2013.04.088, PMID: 23644002

[ref24] Houston J. R. Pollock J. W. Lien M. C. Allen P. A. (2018). Emotional arousal deficit or emotional regulation bias? An electrophysiological study of age-related differences in emotion perception. Exp. Aging Res. 44, 187–205. 10.1080/0361073x.2018.1449585, PMID: 29578840

[ref25] Joyce C. Rossion B. (2005). The face-sensitive n170 and vpp components manifest the same brain processes: the effect of reference electrode site. Clin. Neurophysiol. 116, 2613–2631. 10.1016/j.clinph.2005.07.005, PMID: 16214404

[ref26] Keltner D. Gruenfeld D. H. Anderson C. (2003). Power, approach, and inhibition. Psychol. Rev. 110, 265–284. 10.1037/0033-295X.110.2.265, PMID: 12747524

[ref27] Kraus M. W. Chen S. Keltner D. (2011). The power to be me: power elevates self-concept consistency and authenticity. J. Exp. Soc. Psychol. 47, 974–980. 10.1016/j.jesp.2011.03.017

[ref28] Lang P. J. Bradley M. M. (2010). Emotion and the motivational brain. Biol. Psychol. 84, 437–450. 10.1016/j.biopsycho.2009.10.007, PMID: 19879918 PMC3612949

[ref29] Luo W. Feng W. He W. Wang N. Y. Luo Y. J. (2010). Three stages of facial expression processing: ERP study with rapid serial visual presentation. NeuroImage 49, 1857–1867. 10.1016/j.neuroimage.2009.09.018, PMID: 19770052 PMC3794431

[ref30] Magee J. C. Smith P. K. (2013). The social distance theory of power. Personal. Soc. Psychol. Rev. 17, 158–186. 10.1177/1088868312472732, PMID: 23348983

[ref31] Martin D. Slessor G. Allen R. Phillips L. H. Darling S. (2012). Processing orientation and emotion recognition. Emotion 12, 39–43. 10.1037/a0024775, PMID: 21842989

[ref32] Nissan T. Shapira O. Liberman N. (2015). Effects of power on mental rotation and emotion recognition in women. Personal. Soc. Psychol. Bull. 41, 1425–1437. 10.1177/0146167215598748, PMID: 26231592

[ref33] Paulmann S. Uskul A. K. (2016). Early and late brain signatures of emotional prosody among individuals with high versus low power. Psychophysiology 54, 555–565. 10.1111/psyp.12812, PMID: 28026863

[ref34] Ratcliff N. J. Bernstein M. J. Cundiff J. L. Vescio T. K. (2012a). Seeing wrath from the top (through stratified lenses): perceivers high in social dominance orientation show superior anger identification for high-status individuals. J. Exp. Soc. Psychol. 48, 1373–1376. 10.1016/j.jesp.2012.05.016

[ref35] Ratcliff N. J. Franklin R. G. Jr. Nelson A. J. Vescio T. K. (2012b). The scorn of status: a bias toward perceiving anger on high-status faces. Soc. Cogn. 30, 631–642. 10.1521/soco.2012.30.5.631

[ref36] Recio G. Sommer W. Schacht A. (2011). Electrophysiological correlates of perceiving and evaluating static and dynamic facial emotional expressions. Brain Res. 1376, 66–75. 10.1016/j.brainres.2010.12.041, PMID: 21172314

[ref37] Rellecke J. Sommer W. Schacht A. (2012). Does processing of emotional facial expressions depend on intention? Time-resolved evidence from event-related brain potentials. Biol. Psychol. 90, 23–32. 10.1016/j.biopsycho.2012.02.002, PMID: 22361274

[ref38] Russell A. M. Fiske S. T. (2010). “Power and social perception” in The social psychology of power. eds. Guinote A. Vescio T. K. (New York, NY: Guilford Press), 231–250.

[ref39] Said C. P. Dotsch R. Todorov A. (2010). The amygdala and FFA track both social and non-social face dimensions. Neuropsychologia 48, 3596–3605. 10.1016/j.neuropsychologia.2010.08.009, PMID: 20727365

[ref40] Santesso D. L. Meuret A. E. Hofmann S. G. Mueller E. M. Ratner K. G. Roesch E. B.. (2008). Electrophysiological correlates of spatial orienting towards angry faces: a source localization study. Neuropsychologia 46, 1338–1348. 10.1016/j.neuropsychologia.2007.12.013, PMID: 18249424 PMC2441935

[ref41] Schmid P. C. Kleiman T. Amodio D. M. (2015). Power effects on cognitive control: turning conflict into action. J. Exp. Psychol. Gen. 144, 655–663. 10.1037/xge0000068, PMID: 25893536

[ref42] Schmid Mast M. Jonas K. Hall J. A. (2009). Give a person power and he or she will show interpersonal sensitivity: the phenomenon and its why and when. J. Pers. Soc. Psychol. 97, 835–850. 10.1037/a0016234, PMID: 19857005

[ref43] Singular Inversions (2009). FaceGen Modeller (Version 3.4) [Computer Software]. Toronto, ON: Singular Inversions. Available at: http://facegen.com (Accessed March 10, 2017).

[ref44] Smith P. K. Jostmann N. B. Galinsky A. D. van Dijk W. W. (2008). Lacking power impairs executive functions. Psychol. Sci. 19, 441–447. 10.1111/j.1467-9280.2008.02107.x, PMID: 18466404

[ref45] Uskul A. K. Paulmann S. Weick M. (2016). Social power and recognition of emotional prosody: high power is associated with lower recognition accuracy than low power. Emotion 16, 11–15. 10.1037/emo0000110, PMID: 26414190

[ref46] van Dijk E. van Kleef G. A. Steinel W. van Beest I. (2008). A social functional approach to emotions in bargaining: when communicating anger pays and when it backfires. J. Pers. Soc. Psychol. 94, 600–614. 10.1037/0022-3514.94.4.600, PMID: 18361674

[ref47] Vuilleumier P. (2005). How brains beware: neural mechanisms of emotional attention. Trends Cogn. Neurosci. 9, 585–594. 10.1016/j.tics.2005.10.011, PMID: 16289871

[ref48] Wang J. Liu L. Yan J. H. (2014). Implicit power motive effects on the erp processing of emotional intensity in anger faces. J. Res. Pers. 50, 90–97. 10.1016/j.jrp.2011.05.004

[ref49] Weber H. (2004). Explorations in the social construction of anger. Motiv. Emot. 28, 197–219. 10.1023/b:moem.0000032314.29291.d4

[ref50] Wilkowski B. M. Robinson M. D. (2012). When aggressive individuals see the world more accurately: the case of perceptual sensitivity to subtle facial expressions of anger. Personal. Soc. Psychol. Bull. 38, 540–543. 10.1177/0146167211430233, PMID: 22215701

[ref51] Williams L. M. Palmer D. Liddell B. J. Song L. Gordon E. (2006). The ‘when’ and ‘where’of perceiving signals of threat versus non-threat. NeuroImage 31, 458–467. 10.1016/j.neuroimage.2005.12.009, PMID: 16460966

[ref52] Xu Q. Yang Y. Wang P. Sun G. Zhao L. (2013). Gender differences in preattentive processing of facial expressions: an erp study. Brain Topogr. 26, 488–500. 10.1007/s10548-013-0275-0, PMID: 23371479

[ref53] Yamagishi T. Horita Y. Mifune N. Hashimoto H. Li Y. Shinada M.. (2012). Rejection of unfair offers in the ultimatum game is no evidence of strong reciprocity. Proc. Natl. Acad. Sci. U. S. A. 109, 20364–20368. 10.1073/pnas.1212126109, PMID: 23188801 PMC3528519

